# Secondary Bacterial Infection Rates Among Patients With COVID-19

**DOI:** 10.7759/cureus.22363

**Published:** 2022-02-18

**Authors:** Ilkay Bahceci, Ilknur E Yildiz, Omer F Duran, Umut S Soztanaci, Zeynep Kirdi Harbawi, Feray F Senol, Gokhan Demiral

**Affiliations:** 1 Medical Microbiology, Recep Tayyip Erdoğan University, Medical Faculty, Rize, TUR; 2 Infectious Diseases, Recep Tayyip Erdoğan University, Medical Faculty, Rize, TUR; 3 Anatomy, University of Health Sciences, Istanbul, TUR; 4 Infectious Diseases, Kirikkale Provincial Health Directorate, Kirikkale, TUR; 5 Medical Microbiology, Elazığ Fethi Sekin City Hospital, Elazig, TUR; 6 General Surgery, Recep Tayyip Erdoğan University, Medical Faculty, Rize, TUR

**Keywords:** secondary infection, infection, resistance, covid-19, bacterial infection

## Abstract

Objective

The aim of this study was to determine the factors and rates of secondary bacterial infections developed in patients after the diagnosis of COVID-19 and antimicrobial susceptibility to guide the empirical treatment and contribute to epidemiological data.

Materials and Methods

In our study, 1,055 patients diagnosed with COVID-19, hospitalized at Recep Tayyip Erdoğan University Training and Research Hospital, Rize, between the dates March 24, 2020 and December 31, 2020, were recruited. The diagnoses of all patients were confirmed by positive SARS-CoV-2 polymerase chain reaction (PCR) tests. In addition, the blood and respiratory tract cultures of the patients recruited in the study were analyzed retrospectively.

Results

Ninety-two (8.7%) patients were found to have microbiologically proven respiratory or circulatory tract infections via microbial culture results. Respiratory tract infections were detected as monomicrobial in 44 patients and as polymicrobial in 17 patients, among a total of 61 patients. In addition, 59 (64.1%) patients were male patients, and 33 (35.9%) were female patients. Among the microorganisms grown in blood cultures, *coagulase*-*negative staphylococci* with a percentage of 31% and *Acinetobacter baumannii* with a percentage of 27.5% were prominent. In respiratory tract cultures, *A. baumannii* constitutes the majority with a percentage of 33.3%, followed by *Staphylococcus aureus* and *Klebsiella pneumoniae* with a percentage of 9.5% each. The most resistant bacteria were *A. baumannii*, resistant to all antibiotics other than colistin.

Conclusion

Secondary bacterial infection rates in patients with COVID-19 are lower than influenza pandemic. However, the frequency of empirical antibiotics use seems relatively high.

## Introduction

As of March 2021, COVID-19 is an infectious disease caused by the SARS-CoV-2 virus, which infected over 121 million people worldwide and 2.9 million people in our country, and caused the death of more than 2.6 million people globally and 29,000 people in our country [1]. The clinical symptoms of COVID-19 include a broad spectrum ranging from asymptomatic infection to life-threatening pneumonia [2]. Some patients, particularly those with respiratory distress, are hospitalized and followed up, and severe cases are admitted to ICUs with mechanical ventilation support [3].

The increase in mortality and morbidity due to the susceptibility to infection observed during influenza pandemics has made it essential to question the susceptibility to secondary bacterial infections in patients with COVID-19 [4]. It is difficult to clinically distinguish between bacterial infection and COVID-19 during hospital admission [5, 6]. In addition to this difficulty, the probability of having a secondary bacterial infection among the patients diagnosed with COVID-19 during the clinical course gives rise to the prescriptions of antibiotics to patients with suspected or diagnosed COVID-19 [4]. Although the frequency of secondary bacterial infections among patients infected with SARS-CoV-2 is not known precisely, the negative effects of excessive antibiotic use are of concern [7]. Determining the frequency of secondary bacterial infections among patients, the factors, and the antibiotic susceptibility is important for controlling unnecessary antibiotic use and antibiotic resistance that may occur as a result [4].

In this study, we aimed to contribute to the scientific literature by reporting the prevalence of secondary bacterial infections, etiological agents, and antibiotic resistance among patients who were hospitalized and followed up with the diagnosis of COVID-19 in our hospital.

## Materials and methods

Study group

In our study, 1055 patients diagnosed with COVID-19, hospitalized and followed up at Recep Tayyip Erdoğan University Training and Research Hospital, Rize, between the dates March 24, 2020 and December 31, 2020, were recruited. The diagnoses of all patients were confirmed by positive SARS-CoV-2 polymerase chain reaction (PCR) tests. The blood and respiratory tract cultures of the patients recruited in the study were analyzed retrospectively. Clinical, radiological findings and other infection parameters were not taken into account.

Bacterial culture and antimicrobial susceptibility

The blood cultures of patients sent to the Microbiology Laboratory were incubated in the BacT/ALERT (bioMérieux, France) automated blood culture system. Of the blood culture bottles that detected growth signals, the Gram staining process was conducted, and sheep blood agar, eosin methylene blue (EMB) agar, and chocolate agar were cultivated. The Gram staining process was conducted directly on the samples from lower respiratory tracts of the patients, and sheep blood agar, EMB agar, and chocolate agar were cultivated. Agar plates were incubated in an incubation oven at 35-37°C under aerobic conditions. As a result of 24-48 hours incubation of agar plates, bacterial identification and antibiotic susceptibility of detected colonies were performed with VITEK 2 Compact automated system (bioMérieux, France).

In blood cultures, detecting either a single positive blood culture for probable pathogens or two or more positive blood cultures for skin microbiota (e.g., coagulase-negative staphylococci, viridans group streptococci, diphtheroids, Bacillus spp., Propionibacterium spp.) was defined as “circulatory tract infection.” Repeated culture positivity with the same microorganism was neglected. Antimicrobial sensitivity was analyzed according to the European Committee on Antimicrobial Susceptibility Testing (EUCAST) 2020 criteria.

Culture positivity of probable pathogens in samples from the lower respiratory tract was defined as “respiratory tract infection.” Repeated culture positivity with the same microorganism was neglected.

We accepted that there were no secondary bacterial infections among the patients who did not have any blood and respiratory tract culture orders.

SARS-CoV-2 PCR

According to the manufacturer's instructions, viral nucleic acid extraction was performed from the combined nasopharyngeal and oropharyngeal samples, which were obtained by swab and sent to the laboratory under appropriate transport conditions. Using Bio-Speedy SARS-CoV-2 (2019-nCoV) RT-qPCR Detection Kit (Bioeksen, Istanbul, Turkey) and Coronex COVID-19 rt-qPCR Detection Kit (DS Bio and Nano Technology, Ankara, Turkey), patients' samples were tested according to the manufacturer's instructions. Rotor-Gene Q (QIAGEN, Hilden, Germany) device was used for the tests. Evaluation of the tests was carried out according to the manufacturer's instructions.

## Results

Among 1055 patients included in the study, 92 (8.7%) patients were found to have respiratory or circulatory tract infections, proven microbiologically with culture results. Circulatory infection was detected in 69 of the patients in the study group, 56 of which were monomicrobial and 13 of which were polymicrobial. Respiratory tract infection was detected in 61 individuals, 44 of them were monomicrobial, and 17 of them were polymicrobial (Table 1).

**Table 1:** Distribution of bacterial infections.

	Monomicrobial (n (%))	Polymicrobial (n ( %))	Total (n (%))
Blood culture	56 (81%)	13 (19%)	69 (100%)
Lower respiratory tract culture	44 (72%)	17 (28%)	61 (100%)

Among these 92 patients, 31 patients had only circulatory tract infection, and 23 had only respiratory tract infection. In contrast, both circulatory tract infection and respiratory tract infection were simultaneously observed in 38 patients.

Of the patients with infection, 59 patients (64.1%) were male with an average age of 71.9 years, and 33 patients (35.9%) were female with an average age of 70.8 years. While 73 (79.3%) of the patients were hospitalized at ICUs, 19 of them (20.6%) were patients hospitalized at inpatient wards.

**Figure 1:**
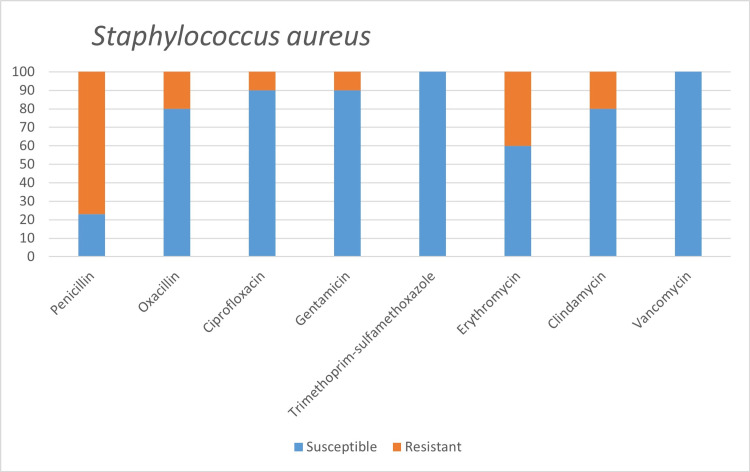
Antimicrobial susceptibility of Staphylococcus aureus.

**Figure 2:**
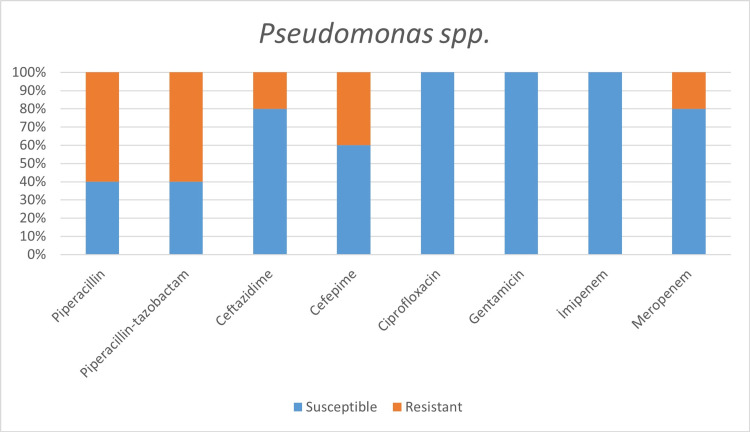
Antimicrobial susceptibility of Pseudomonas spp.

**Figure 3:**
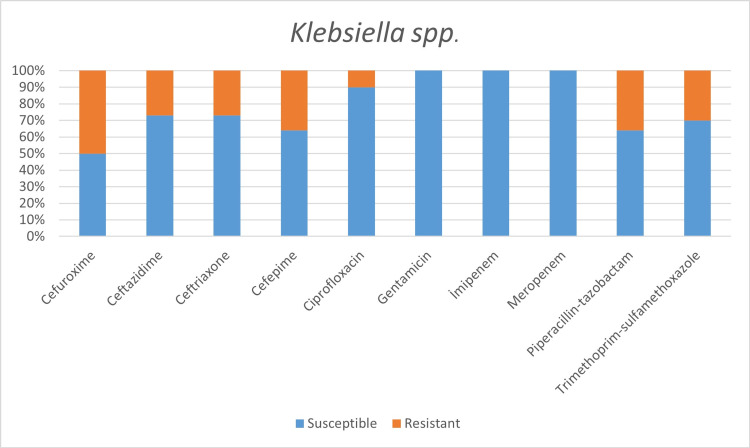
Antimicrobial susceptibility of Klebsiella spp.

**Figure 4:**
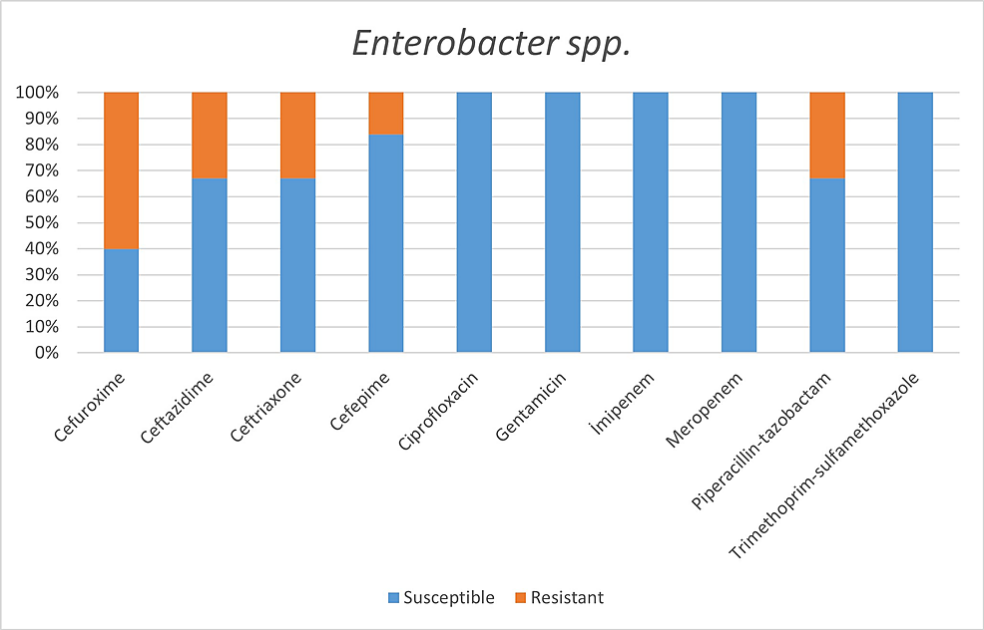
Antimicrobial susceptibility of Enterobacter spp.

**Figure 5:**
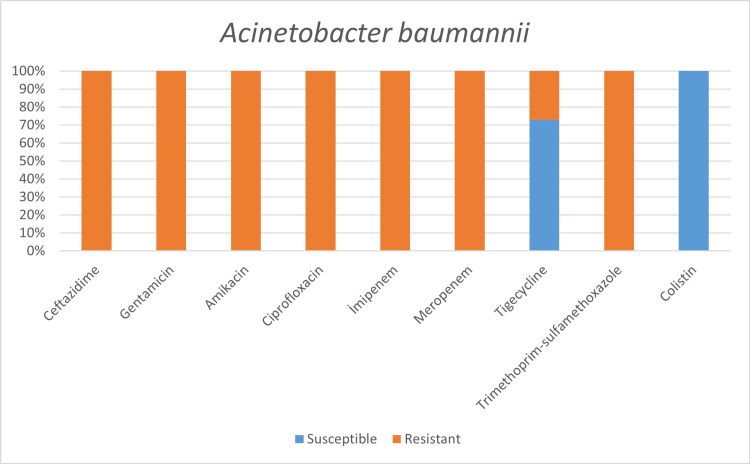
Antimicrobial susceptibility of Acinetobacter baumannii.

**Figure 6:**
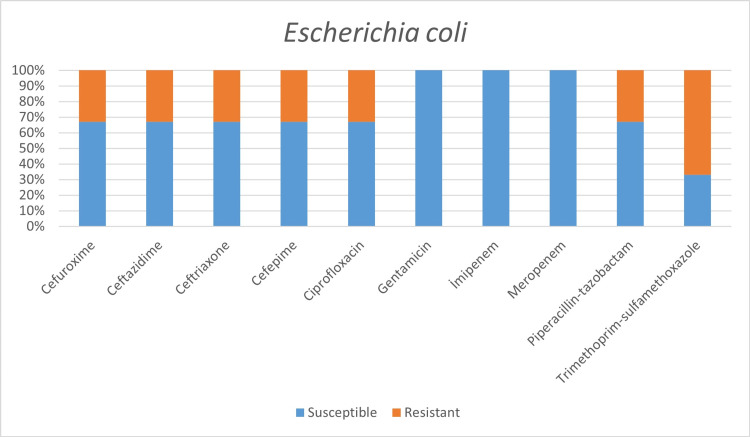
Antimicrobial susceptibility of Escherichia coli.

Among the microorganisms grown on blood cultures, coagulase-negative staphylococci with a percentage of 31% and *A. baumanni*i with a percentage of 27.5% were prominent. In respiratory tract cultures, *A. baumannii* constitutes the majority with a percentage of 33.3%, followed by *S. aureus* and *K. pneumoniae* with a percentage of 9.5% each (Table 2). The status of antimicrobial susceptibility of the bacteria that have grown frequently is given in Figures 1-6.

**Table 2:** Bacterial infection agents.

	Blood culture (n (%))	Lower respiratory tract culture (n (%))	Total (n (%))
Acinetobacter baumannii	24 (14%)	28 (16.3%)	52 (30.4%)
Staphylococcus aureus	2 (1.1%)	8 (4.6%)	10 (5.8%)
Klebsiella spp.	2 (1.1%)	9 (5.2%)	11 (6.4%)
Escherichia coli	2 (1.1%)	1 (0.5%)	3 (1.7%)
Enterobacter spp.	2 (1.1%)	4 (2.3%)	6 (3.5%)
Coagulase-negative staphylococci	27 (15.7%)	0	27 (15.7%)
Pseudomonas aeruginosa	1 (0.5%)	2 (1.1%)	3 (1.7%)
Enterococcus spp.	10 (5.8%)	1 (0.5%)	11 (6.4%)
Other	17 (9.9%)	31 (18.1%)	48 (28%)
Total	87 (50.8%)	84 (49.1%)	171 (100%)

## Discussion

We detected microbiologically documented secondary bacterial infections in 92 (8.7%) of 1055 COVID-19 patients that we examined in our study. This rate shows that more than 30% of secondary bacterial infections detected in previous influenza pandemics are lower in COVID-19 disease [8]. Similarly, Lansbury L et al. found that secondary bacterial infection observed in COVID-19 patients was less common than in influenza patients, as a result of their meta-analysis [9].

Through the meta-analyses performed to determine the secondary bacterial infection rates among COVID-19 patients, Langford BJ et al. [4] found the rate of 6.9%. In contrast, Rawson TM et al. [10] found it as 8% and Lansbury L et al. [9] as 7%. Within the studies investigating ICUs, this rate has increased to 14% [9]. Furthermore, when COVID-19 patients died, they were retrospectively examined, and it was observed that the prevalence rates of secondary bacterial infections could reach up to 50% [11]. Therefore, the rate of secondary bacterial infection that we detected as 8.7% in our study also shows parallelism with the meta-analyses mentioned above.

Secondary bacterial infections mainly occur due to the use of mechanical ventilators and catheters among intensive care patients [12]. Thus, the fact that 79.3% of the patients in whom we detected microbial growth are intensive care patients supports this situation. Although immunosuppressive treatments and hospitalization paved the way for infection, it is likely that the increase in isolation measures and the high rate of empirical antibiotic use were effective in keeping the secondary bacterial infection rate low [12]. Despite the low incidence of secondary bacterial infections, studies show that over 90% of COVID-19 patients are prescribed antibiotics [13, 14]. This increase in antibiotic use creates a basis for infection with resistant bacteria by increasing antimicrobial resistance [15]. More strikingly, in a study conducted in Wuhan, antibiotic use was associated with higher rates of in-hospital deaths [16]. In parallel with the higher incidence of secondary bacterial infections among critically ill patients, the WHO guidelines recommend empirical antibiotic use only in severe COVID-19 patients [17, 18].

The incidence rates of microorganisms vary between studies. When respiratory tract infections are investigated, besides the studies reporting *A. baumannii* as the most common secondary bacterial infection agent, the studies reporting S. aureus [7, 17] and Pseudomonas aeruginosa [2] as the most common secondary bacterial infection agents are also included in the literature. The bacterium identified as another opportunistic pathogen for the respiratory tract is Stenotrophomonas maltophilia [19]. In our study, *A. baumannii* emerged as the most frequently isolated agent in respiratory tract infections, in parallel with the fact that the majority of patients who developed secondary bacterial infections were intensive care patients. S. maltophilia's microbial growth was not detected at all.

When we look over circulatory infections, apart from studies reporting coagulase-negative staphylococci [2] as the most common secondary bacterial infection agent, there are also studies reporting *S. aureus* [7, 17]. In our study, the most common agents detected in circulatory tract infections were coagulase-negative staphylococci (31%) and *A. baumannii* (27.5%).

One of the aims of our study was to determine the susceptibility to commonly used antibiotics among bacteria, which are common infectious agents in COVID-19 patients. The empirically initiated antibiotics and the susceptibility of detected infectious agents to that antibiotics hold great importance. In the study of Vaughn VM et al. [20], it was reported that the most commonly prescribed antibiotics were ceftriaxone 38.9%, vancomycin 13.8%, and cefepime 10.4%. In addition, while 63.4% of the patients received empirical antibiotics for community-acquired agents, only 26.3% received antipseudomonal, and 25.8% received antimicrobial therapy effective against methicillin-resistant Staphylococcus aureus (MRSA) [20].

Resistant bacteria, mainly *A. baumannii*, seem to be quite common among hospitalized patients and pose a significant problem for health systems [3]. In addition to the fact that *A. baumannii* was the most common agent in our study, we found high antimicrobial resistance rates against *A. baumannii*, except for colistin and tigecycline. For example, while all of our *A. baumannii* strains showed susceptibility to colistin, Sharifipour E et al. found 52% resistance to colistin and a high level of resistance to other antibiotics in a study conducted in Iran [3].

In a study conducted by Mahmoudi H [7] with COVID-19 patients, he revealed the highest resistance in Enterobacteriaceae isolates to trimethoprim/sulfamethoxazole 74%, piperacillin 67.5%, ceftazidime 47.5%, and cefepime 42.5%. The highest resistance rates we detected in the Enterobacteriaceae family were revealed as: 

Within E. coli isolates: trimethoprim/sulfamethoxazole 67%, cefuroxime 33%, ceftriaxone 33%, cefepime 33%, ciprofloxacin 33%; 

Within Klebsiella spp. isolates: cefuroxime 50%, cefepime 36%, piperacillin/tazobactam 36%; 

Within Enterobacter spp. isolates: cefuroxime 60%, ceftazidime 33%, ceftriaxone 33%, piperacillin/tazobactam 33%. 

While 8.6% of the isolates belonging to the Enterobacteriaceae family were imipenem-resistant, all isolates were observed to be susceptible to imipenem.

Staphylococcus aureus is a common cause of secondary bacterial infection in influenza pandemics. This is considered to be related to the bacteria's ability to attach to the respiratory mucosa [21]. Methicillin resistance is vital within *S. aureus* strains, and studies are showing that lower respiratory tract infections caused by MRSA are more mortal. Methicillin resistance was observed in 2 (20%) of 10* S. aureus* isolates detected in our study. In another study conducted among COVID-19 patients, 15 *S. aureus* isolates, 40% of which were reported as MRSA, were detected [7].

Study limitations

All infections detected after the hospitalization of the patients were considered secondary bacterial infections. No distinction was made between secondary bacterial infection and coinfection. Only laboratory parameters were examined without focusing on the clinical and radiological data of the patients. The survival status of the patients as a result of their clinical course was not included in our study. Only lower respiratory tract and circulatory tract infections were included within the scope of secondary bacterial infection. In addition, the resistance genes of the isolated bacterial agents were not studied.

## Conclusions

It has been determined that coagulase-negative Staphylococci were the most common in blood circulation infections and *A. baumannii* respiratory tract infections. All *S. aureus spp.* were resistant to methicillin. *Enterobacteriaceae* family was susceptible to imipenem and meropenem, and *A. baumannii* was susceptible to colistin and tigecycline. However, the use of empirical antibiotics is relatively high. This situation poses a threat to antimicrobial resistance, both now and in the future, and increases healthcare costs. 
